# Exploring the Role of Vitamin D. Comments on Fleury et al. Sun Exposure and Its Effects on Human Health: Mechanisms through Which Sun Exposure Could Reduce the Risk of Developing Obesity and Cardiometabolic Dysfunction. *Int. J. Environ. Res. Public Health* 2016, *13*, 999

**DOI:** 10.3390/ijerph13121256

**Published:** 2016-12-18

**Authors:** Barbara J. Boucher, William B. Grant, Harjit Pal Bhattoa

**Affiliations:** 1 The Blizard Institute, Barts and the London School of Medicine & Dentistry, Queen Mary University of London, London E12AT, UK; b.j.boucher@qmul.ac.uk; 2 Sunlight, Nutrition, and Health Research Center, P.O. Box 641603, San Francisco, CA 94164, USA; wbgrant@infionline.net; 3 Department of Laboratory Medicine, Faculty of Medicine, University of Debrecen, Nagyerdei Blvd 98, Debrecen H-4032, Hungary

The mechanistic data presented in this interesting review suggests that long-term exposure to safe levels of ultra-violet radiation (UVR) has protective effects against the development of obesity and cardiovascular dysfunction beyond those induced by the cutaneous synthesis of vitamin D3 through factors such as the induction of cutaneous NO secretion [1]. Evidence from studies achieving comparable vitamin D status (serum 25-hydroxyvitamin D (25OHD) concentrations) is quoted [2], where features of metabolic syndrome were better suppressed by the UVR doses used than by supplementation. However, higher vitamin D status has been shown to have similar beneficial effects, for example on serum lipids [3], even without weight loss, and on insulin resistance [4]. Since homeostatic mechanisms come into effect both in the skin, and systemically, as vitamin D provision increases [5], it would be helpful to the authors’ arguments if UVR treated mice did not show evidence of increased homeostatic mechanism activity in comparison with the supplemented animals, despite the comparable achieved 25OHD findings. For example, in comparing data from animals treated with UVR, can the authors tell us whether or not serum parathyroid hormone values achieved were lower, or whether the serum calcium or serum calcitriol contents were higher than they were in the supplemented group? The absence of differences in these bio-markers of vitamin D provision and activity after treatment with UVR or vitamin D supplementation, where comparable serum 25OHD concentrations were achieved, would strengthen the authors’ arguments considerably.
